# Global Proteomics Reveals Distinct Muscle Adaptations to Menstrual Cycle Phase-Based Sprint Interval Training in Endurance-Trained Females

**DOI:** 10.1016/j.mcpro.2025.101053

**Published:** 2025-08-14

**Authors:** Julie Kissow, Kamine Julie Jacobsen, Søren Jessen, Laura Bachmann Thomsen, Júlia Prats Quesada, Jens Bangsbo, Atul Shahaji Deshmukh, Morten Hostrup

**Affiliations:** 1 The August Krogh Section for Human Physiology, Department of Nutrition, Exercise and Sports, University of Copenhagen, Copenhagen, Denmark; 2 The Novo Nordisk Foundation Center for Basic Metabolic Research, Faculty of Health and Medical Sciences, University of Copenhagen, Copenhagen, Denmark

**Keywords:** estrogen, exercise, female, performance, sex hormones, athletes

## Abstract

Advances in mass-spectrometry (MS)-based technologies have leveraged our understanding of protein-wide adaptations in human skeletal muscle in response to exercise. However, there is a lack of such data in females, particularly pertaining to already trained females and menstrual cycle phase-based sprint interval training (SIT) despite its efficacy and popularity. Here, we present a comprehensive global proteome analysis of skeletal muscle adaptations to high-frequency SIT during different menstrual cycle phases in endurance-trained females. We randomized 49 eumenorrheic females to either high-frequency SIT in the follicular (FB) or luteal phase (LB) over one menstrual cycle comprising eight sessions of 6 × 30-s all-out efforts. MS-proteomics, covering 4155 proteins after filtering, revealed notable differences in muscle adaptations to phase-based SIT. LB suppressed mitochondrial pathways of the tricarboxylic acid cycle and electron transport chain while enriching ribosomal complexes. Conversely, FB enriched filament organization and skeletal system development. Mitochondrial repression during LB was linked to reduced $\dot{V}$ O_2max_, whereas exercise capacity improved in FB only. Our findings show that menstrual cycle phase-based high-frequency SIT induces distinct protein-wide muscle adaptations and affects phenotype in endurance-trained eumenorrheic females. NCT04136457.

## Highlights

• First to assess the global muscle proteome in females across menstrual phases.
• MS-proteomics reveals phase-specific muscle adaptations to sprint interval training.
• Luteal phase-based sprint interval training suppresses mitochondrial pathways.
• Follicular phase-based sprint interval training enhances filament organization.
• Phase-based programming may optimize training outcomes in well-trained females.

## Graphical Abstract

**Figure:**
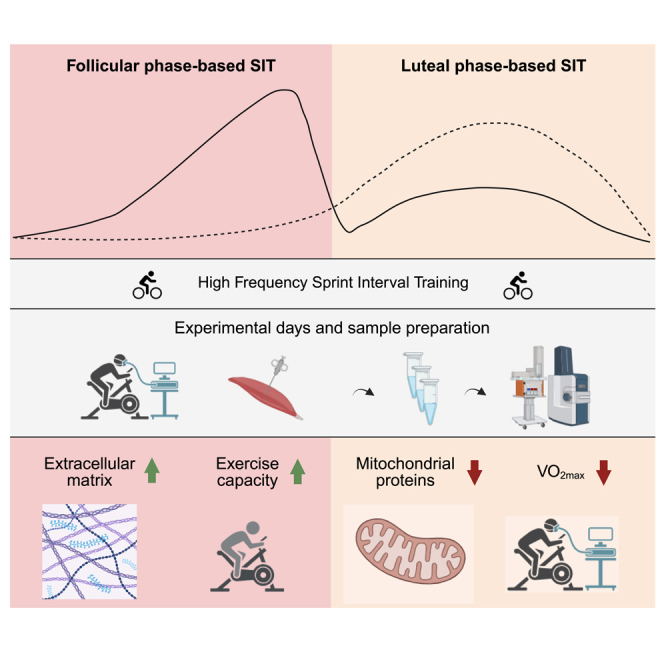

## In Brief

This study provides the first comprehensive proteomic analysis of menstrual cycle phase-based sprint interval training (SIT) in endurance-trained females. High-frequency SIT during the follicular phase preserved mitochondrial function and enhanced tissue remodeling, while luteal phase-based training suppressed mitochondrial pathways and reduced cardiorespiratory fitness. These findings emphasize the importance of considering menstrual cycle phase in female training strategies.

Skeletal muscle plays a critical role in driving the health- and performance-related benefits of exercise (1). It exhibits a remarkable ability to adapt in response to even short periods of physical activity (2, 3). Recent advances in mass-spectrometry (MS)-based technologies have leveraged the muscle physiological field by allowing in-depth coverage of thousands of proteins. Harnessing MS-proteomics, different research groups have revealed that hundreds of proteins in human skeletal muscle respond differentially to only a few weeks of exercise training (4, 5, 6, 7, 8). However, all these studies were performed with male participants only. Investigating global protein-level adaptations in female participants would provide valuable insights and could reveal distinct regulation patterns (9, 10). One such aspect pertains to the impact of menstrual cycle phases on protein-wide muscle adaptations to sprint interval training (SIT), which is an increasingly popular and effective training form (11, 12, 13).

Ever since Gamberale *et al*. demonstrated in 1975 that pulmonary ventilation during exercise fluctuates throughout the menstrual cycle, there has been growing interest in understanding how variations in female sex hormones impact performance and training responsiveness (14, 15, 16). In the early-follicular phase, estrogen and progesterone levels are low, contrasting with the late-follicular phase marked by increased estrogen levels leading up to ovulation. Subsequently, in the luteal phase, both hormones are secreted (17). Some evidence suggests that estrogen has an anabolic effect on skeletal muscle and thus can lead to an increased potential for training adaptations (18, 19, 20). In accordance, a few studies found superior resistance training adaptations to high-frequency training during the follicular phase compared to the luteal phase in terms of enhancing muscle strength and mass (15, 16). However, knowledge about intrinsic protein-wide adaptations within skeletal muscle is absent. But given the greater effects of high-frequency resistance training in the follicular than the luteal phase, it is conceivable that differential protein-wide muscle adaptations occur that drive these functional differences.

Here, we employ a comprehensive MS-based proteomics workflow and a strict set of inclusion criteria with longitudinal monitoring in a cohort of endurance-trained females to uncover protein-wide muscle adaptations in response to high-frequency SIT in either the follicular or luteal phase of the menstrual cycle. We hypothesized that phase-based SIT would induce differential protein-wide adaptations, particularly pertaining to oxidative metabolism, with superior effects of high-frequency SIT in the follicular phase when compared to the luteal phase for $\dot{V}$ O_2max_.

## Materials and Methods

### Ethics Approval

The study was conducted in accordance with the standards set by the 2013 version of the Declaration of Helsinki and was approved by the regional ethics committee of Copenhagen, Denmark (H-20052639).

### Study Goals and Design

**Fig. 1:**
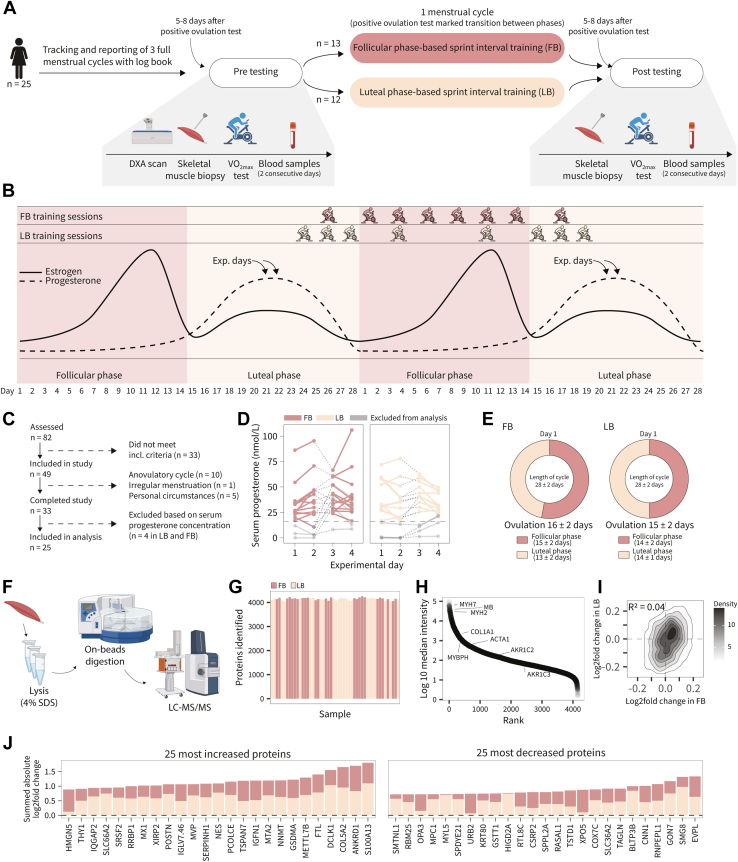
**High-frequency phase-based sprint interval training (SIT) in the follicular phase (FB) and luteal phase (LB) induces differential muscle proteome remodeling.***A*, experimental overview of the study design. Participants tracked their menstrual cycle for three cycles prior to enrollment to ensure a regular menstrual cycle (between 21 and 35 days). *B*, overview of the menstrual cycle phase-based sprint interval training intervention. *C*, flow diagram of the inclusion process. *D*, serum progesterone concentrations on trial days. The lower limit on 16 nmol L^−1^ is indicated by a dashed line, and participants who were below this limit on both occasions were excluded from data analysis (marked by *grey* lines and symbols). Participants are marked by dashed lines between symbols between Trials 2 and 3. *E*, Mean length for the menstrual cycle in the two groups. *F*, overview of sample preparation. *G*, protein groups identified per sample. *H*, dynamic range of protein identifications. *I*, correlation density plot between log2fold changes of each protein in FB and LB with Pearson correlation coefficient. *J*, summed mean log2fold changes (absolute values) of the top 25 increased and decreased proteins with the training interventions irrespective of adjusted *p*-value. Full results are available in Supplementary Data 2. DXA, Dual Energy X-Ray Absorptiometry; FB, follicular phase-based training; LB, luteal phase-based training.

The study was a longitudinal, randomized parallel-group study, and aimed to clarify how high-frequency phase-based SIT during either the follicular or luteal phase of the menstrual cycle influences performance and skeletal muscle proteome remodeling in trained females. Of the 82 females initially screened, 49 met the inclusion criteria and were randomized to either the follicular or the luteal phase-based training group. A total of 33 completed the intervention of which eight were excluded before the final analysis due to serum progesterone levels below 16 nmol/L. A detailed participant flowchart is shown in Figure 1*C*.

All trials were conducted at the Department of Nutrition, Exercise and Sports, University of Copenhagen, Denmark, between October 2020 and July 2022. All participants were informed about the possible risks involved and gave their oral and written consent before the conclusion. Inclusion criteria were healthy eumenorrheic trained females not using hormonal contraceptives and with a regular menstrual cycle, aged 18 to 45 years. $\dot{V}$ O_2max_ ≥ 50 ml∙kg^−1^ min^−1^ (≤10% due to biological and technical variation) and ≥3 training sessions weekly. The included females performed primarily running, cycling, swimming, and CrossFit. Exclusion criteria were chronic disease, injury on the musculoskeletal system, smoking, menstrual cycle >35 days, luteal phase-deficiency, anovulatory cycles, or pregnancy.

#### Assessment of Eligibility Criteria

In the current study, we defined a regular menstrual cycle based on the following criteria: a menstrual cycle between 21 to 35 days (14, 21), positive ovulation, and serum progesterone concentrations above 16 nmol/L (14). To verify these criteria and to track the transition between the follicular and luteal phase, we utilized a comprehensive inclusion process (Fig. 1*A*). All females reported the length of three full menstrual cycles before the intervention. In addition, previous users of hormonal contraceptives (four to 6 months prior) needed a positive ovulation test before baseline measurements to prevent inclusion of females with anovulatory cycles. After baseline measurements, all included participants tracked two full menstrual cycles by calendar-based counting and an ovulation test. The onset of menses was registered as day 1, which indicated the change from luteal to follicular phase, and the day of a positive ovulation test confirmed the transition from the follicular to luteal phase. (Fig. 1*B*). The participants started ovulation testing from Day 9 of the menstrual cycle until a positive test result occurred, which determined the scheduling of the subsequent trial period for the individual participant. Furthermore, we quantified serum progesterone concentrations during experimental days and excluded participants with values below 16 nmol L^−1^ from data analysis, as this can indicate luteal phase deficiency (14).

We assessed $\dot{V}$ O_2max_ by an incremental test to exhaustion on a bicycle ergometer (Lode Excalibur Sport, 10.10.1, The Netherlands, Groningen) using an online breath-by-breath gas analyser system (Oxycon Pro, Viasys Healthcare). The incremental test was preceded by a 12-minute lead-in period of 4 min at 75, 100, and 125 W, respectively, followed by an increase in resistance by 25 W·min^−1^ until exhaustion. Incremental peak power output (iPPO) was defined as the highest workload (W) during the incremental test. After a recovery period of 10 min, participants had a 30-s lead-in period of 75 W, followed by an increase in workload to 110% of iPPO until exhaustion to maximize the likelihood of achieving true $\dot{V}$ O_2max_. The pre-incremental test for $\dot{V}$ O_2max_ served as a baseline to confirm participants met the inclusion criteria, although its timing varied across menstrual phases. However, this was not expected to affect results, as prior studies indicate $\dot{V}$ O_2max_ and iPPO remain stable throughout the menstrual cycle in moderately trained and trained females (22, 23, 24, 25, 26, 27).

### Outcome Measurements and Cohort Size

The main outcome measure was exercise performance as determined during a ramp test to exhaustion. Other outcomes were $\dot{V}$ O_2max,_ and muscle protein adaptations as investigated by MS-based proteomics and immunoblotting. Sample size calculations were performed using GPower 3.1.9.3 for the primary outcome (exercise performance), assuming a medium effect size (Cohens d = 0.5) with an α-level of 0.05 and β-level of 0.8 for a linear mixed model repeated measures design. This resulted in a required sample size of 12 participants per group.

### Experimental Days

If all inclusion criteria and none of the exclusion criteria were met, participants completed a pre-trial day. At this pre-trial day (placed 1–5 days after positive ovulation test), a muscle biopsy was collected at rest from the vastus lateralis muscle using a Bergström biopsy needle through an incision made in the skin under local anesthesia (20 mg·mL-1 Xylocaine; AstraZeneca). Muscle biopsies were immediately rinsed in ice-cold saline (9 mg/ml, Fresenius Kabi, Sweden) and dissected free of visible fat, blood, and connective tissue before being frozen in liquid nitrogen and stored at −80 °C for later analysis.

Two experimental days were conducted pre- and post-training intervention, placed 5 to 8 days after positive ovulation (mid-luteal phase) (Fig. 1*A*). On the first pre-experimental day, body composition was determined by dual-energy X-ray absorptiometry (DXA; Lunar iDXA, GE Healthcare, GE Medical Systems). Furthermore, on both pre-experimental days, a blood sample was collected from the brachial vein in a 3.5 ml serum separator tube to measure serum progesterone concentration. The blood sample was analyzed at Clinical Biochemistry Department 3011, Rigshospitalet. After the pre-intervention experimental days, participants were randomized, stratified for $\dot{V}$ O_2max_ using minimization, to either high-frequency SIT in the follicular phase (FB) or luteal phase (LB) lasting the length of one menstrual cycle for the individual participant (28 ± 2 days). 48 to 72 h after completing the training intervention, participants completed two experimental days consisting of blood samples, as described during experimental days prior to the training intervention.

### Training Intervention

The training intervention consisted of eight SIT sessions on a spinning bike (Body Bike Indoor Cycle, W014) (Fig. 1*B*). FB performed high-frequency SIT in the follicular phase (6 training sessions) and low frequency SIT in the luteal phase (2 training sessions). In contrast, LB performed low frequency SIT in the follicular phase (2 training sessions) and high-frequency SIT in the luteal phase (6 training sessions) (Fig. 1*B*). A training session consisted of a 5-min warm-up period at a self-selected resistance. Then, 6 × 30-s all out bouts interspersed by 3 min of rest were performed on a spinning bike. At the last training session, a biopsy was collected from the m. vastus lateralis at rest. Subsequently, a $\dot{V}$ O_2max_-ramp test to exhaustion was performed on a cycle ergometer, and participants completed a training session on a spinning bike consisting of 5 × 30-s all out bouts interspersed by 3 min of rest. In FB, participants completed 7 (n = 2) or 8 (n = 11) training sessions over 28 ± 2 days, while in LB, participants completed 7 (n = 3) or 8 (n = 9) training sessions over 28 ± 2 days. All missed or misplaced training sessions were attributed to menstrual cycle variations. The training intervention was implemented in addition to each participant’s habitual training. All participants were instructed to maintain their usual training routines throughout the intervention period. This was done to ensure that any observed changes in performance or muscle adaptations could be attributed specifically to the SIT intervention, rather than to reductions or modifications in their normal weekly training volume or intensity. This approach was taken to preserve ecological validity and minimize confounding effects related to detraining or altered exercise behavior.

### Subject Source and Description

This study focused on physiological adaptations in healthy, trained females and did not include differential-diagnostic control groups, as it was not designed to evaluate diagnostic biomarkers. Participants were free from chronic disease and known confounders such as smoking, hormonal contraceptive use, or pregnancy. To minimize intra-individual hormonal variability, all testing and sampling were precisely timed based on ovulation tests and serum progesterone levels. As the cohort consisted of healthy individuals, the International Classification of Diseases (ICD) and disease-specific staging were not applicable.

Following pre-intervention experimental days, participants were randomized using a block randomization scheme to either follicular or luteal phase-based high-frequency SIT intervention. Randomization was stratified by $\dot{V}$ O_2max_ (mL∙kg^−1^ min^−1^) to ensure balanced baseline fitness levels between groups.

Muscle biopsies and blood samples were obtained at the Department of Nutrition, Exercise and Sports, University of Copenhagen, by trained personnel and processed using standardized protocols to ensure consistency and sample integrity.

## Experimental Procedures

### Proteomic Sample Preparation

Freeze-dried muscle tissue was finely dissected under a stereomicroscope to remove all visible fat, blood, and connective tissue. The powdered tissue was then lysed with 4% sodium dodecyl sulfate (SDS), 100 mM Tris at pH 8.5, using a BeatBox homogenizer (Preomics). The resulting lysate was immediately boiled for 5 min, cooled on ice, and subjected to tip-probe sonication (30:30s on/off, 15 cycles). Samples were centrifuged at 16,000*g* for 10 min, and the supernatant obtained was used for protein concentration determination employing a bicinchoninic acid (BCA) assay. For further processing, 50 μg of protein lysate was reduced with 10 mM tris (2-carboxyethyl) phosphine (TCEP) and alkylated with 40 mM 2-chloroacetamide (CAA), for 5 min at 40 °C. An overnight on-bead digestion using a combination of trypsin (1:100 enzyme: protein) and lysC (1:500 enzyme: protein) was carried out via protein aggregation capture (PAC) on a KingFisher Flex robot (Thermo), as previously described (28). Protein digestion was quenched by adding 1% trifluoroacetic acid (TFA), and the peptides were desalted on C18 cartridges (Sep-Pak, Waters). Peptides were eluted with 50% acetonitrile (ACN), followed by vacuum drying to determine the concentration by measuring 280/260 absorbance in a NanoDrop spectrophotometer (Thermo Scientific). Finally, 200 ng of peptides were loaded onto Evotips previously equilibrated and ready for measurement.

### Mass Spectrometry Analysis

The samples underwent separation using a 15-cm column with a diameter of 150 μm, packed with C18 beads (1.5 μm, Pepsep), on an Evosep ONE HPLC. The system utilized the default 30-SPD method, allowing for the processing of 30 samples per day, with the column temperature maintained at 50 °C. After elution, peptides were injected into a timsTOF Pro 2 mass spectrometer (Bruker) using a CaptiveSpray source and a 20-μm emitter, operating in diaPASEF mode (29). For the global proteome profiling, the mass spectrometry data covered a range from 100 to 1700 m/z. During the collection of MS/MS data, each diaPASEF cycle lasted 1.8 s, covering an ion mobility range of 1.6-0.6 1/K0. The diaPASEF method employed a long-gradient approach, consisting of 16 diaPASEF scans with two 25 Da windows per ramp, setting a mass range of 400.0-1201.0 Da and a mobility range of 1.43-0.60 1/K0. Collision energy varied according to ion mobility and decreased linearly from 59 eV at 1/K0 = 1.3 to 20 eV at 1/K0 = 0.85 Vs cm_2_. Calibration of ion mobility was performed using three Agilent ESI-L Tuning Mix ions (m/z 622.0289, 922.0097, and 1221.9906) with both accumulation and PASEF ramp times constant at 100 ms.

### Experimental Design and Statistical Rationale

#### Mass Spectrometry Acquisition

The study included 25 subjects, each with pre- and post-intervention samples. Mass spectrometry data were acquired using a data-independent acquisition (DIA) strategy and analyzed in Spectronaut (v18.6, Biognosys) using the library-free directDIA + workflow. A project-specific spectral library was generated in real time from all 50 biological samples; no external or public libraries were used. MS1 data acquisition was performed, and no retention time or spiked peptide standards were included.

DIA fragmentation employed dynamic mass tolerance (threshold 1), with the number of DIA windows optimized automatically by Spectronaut. Thirty-two ion mobility windows were acquired (range: 0.751–1.281), with overlapping determined by the acquisition method. The total cycle time for MS1 and MS2 scans was 1.80768 s.

Samples were loaded in alternating group-wise order, to mitigate batch effects. A 1% false discovery rate (FDR) threshold was used at the peptide-spectrum match (PSM), peptide, and protein group levels to ensure statistical robustness.

#### Mass Spectrometry Data Processing

Raw DIA data were searched against the SwissProt human reference FASTA (January 2023, 20,404 protein entries) using Spectronaut’s directDIA + workflow. Trypsin/P digestion was performed, allowing up to two missed cleavages. Carbamidomethylation (Cys) was set as a fixed modification, while oxidation (Met) and N-terminal acetylation were defined as variable modifications.

Data processing incorporated precursor signal alignment using dynamic XIC extraction (set to 1) and m/z extraction based on maximum intensity. Fragment ion selection was intensity-based, utilizing DNN-predicted ion mobility (set to automatic). Fragment ions were filtered based on amino acid length (minimum: 3), m/z range (200–3000), and a relative intensity threshold (minimum: 1). Precursor filtering selected the best N fragments per peptide (minimum: 3, maximum: 6).

FDR thresholds of 1% were applied at the PSM, peptide, and protein group levels. Decoys were generated using a mutated approach, with a dynamic decoy limit set at 10% of the library size. These decoy entries were used for protein-level FDR estimation. Protein grouping followed Spectronaut’s default “Single Protein per Gene” strategy.

Quantification was based on major group precursor intensities, with local normalization applied at the precursor level. Only peptides passing the 1% FDR threshold were included in quantification. Retention time alignment was automatically optimized. Ion mobility separation used a dynamic ion mobility peak filter targeting a TIC fraction of 0.9, and precursor detection was performed dynamically, with mass tolerance set to dynamic (value: 1).

The quantification results from the Spectronaut analysis were obtained using "Area" as the quantity type at the MS2 level, ensuring that intensity values were derived directly from the fragment ions in the MS2 spectra. Quantification was performed using the Automatic method with Cross-Run Normalization enabled, maintaining consistency across multiple runs. No specific imputation strategy was applied during data processing, meaning that missing values were neither estimated nor replaced. Additionally, no lower intensity threshold was set for including precursor data.

To ensure data reliability, protein quantification required a minimum of one peptide, with a maximum of three peptides considered per protein group. This criterion ensured that proteins identified with fewer than one peptide were excluded from the final analysis.

#### Performance Data

The statistical analysis of performance data (i.e., time trial mean power output, $\dot{V}$ O_2max_, and incremental exercise performance) was carried out in SPSS version 28 (IBM Software). Normal distribution of data was confirmed by Q-Q plots and the Shapiro–Wilk test. A 2 × 2 linear mixed-effects model with time (pre/post) and group (FB/LB) as fixed factors and participant as a random factor was used to examine differences within and between groups over the intervention period. Data is presented as mean ± standard deviation, and effect sizes as mean change with ± 95% confidence interval. *p*-values are presented to represent probability.

### Bioinformatics

#### Preprocessing

All bioinformatic analyses were conducted in R (v.4.3.2, Foundation for Statistical Computing, Vienna, Austria). Data was filtered for 70% valid values in at least one group (FB or LB), resulting in 4155 remaining proteins (4303 proteins before filtering). Values were log_2_-transformed and median-scaled, followed by annotation with gene ontology terms (GO:BP, GO:CC, GO:MF) and keywords from the UniProt database. Mitochondrial proteins were annotated with MitoCarta 3.0 (30).

#### Differential Expression Analysis

Statistical analysis of proteomics data was conducted with the limma package (v. 3.52.4) on non-imputed data (31). A linear model was fit using protein-wise log_2_-transformed intensities, incorporating both time (pre/post) and intervention group (FB/LB) as fixed effects. Individual participant ID was included as a blocking factor to account for within-subject correlation using limma::duplicateCorrelation. All models were also run with the inclusion of selected covariates (baseline bodyweight, $\dot{V}$ O_2max_, and weekly training hours) which differed at baseline. The effects of covariate inclusion are described in Supplementary data S5. Contrasts were specified to evaluate the main effect of time (post vs. pre) and within-group post vs. pre changes (FB and LB separately). Models were fit using limma::lmFit, and statistical inference was performed using empirical Bayes moderation (limma::eBayes). To control for multiple testing, the false discovery rate method described by Storey and Tibshirani (32) was employed with the R package “qvalue” (v.2.28.0). Individual protein results, including raw and adjusted *p*-values (33), are available in Supplementary data S2.

#### Gene Set Enrichment Analysis

**Fig. 2:**
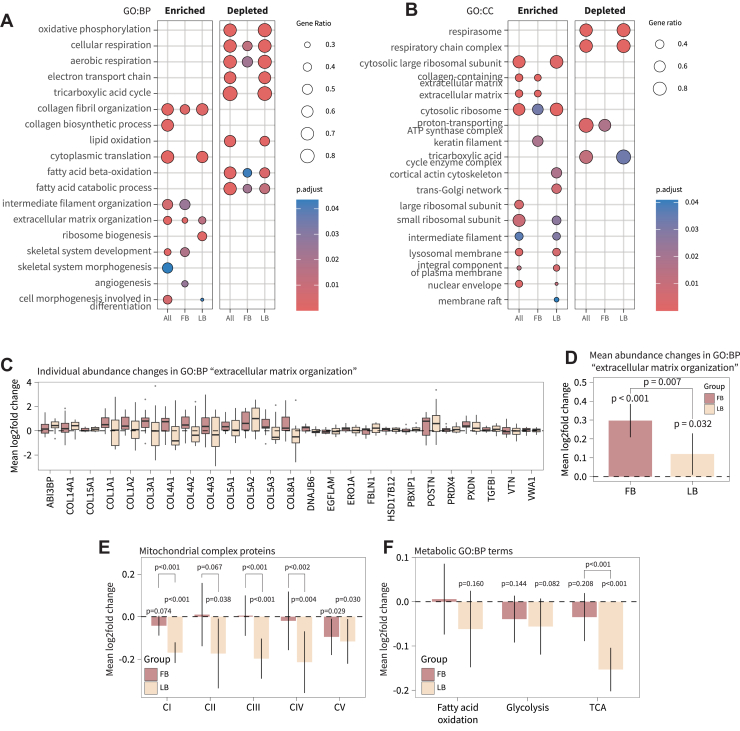
**Four weeks of high-frequency phase-based SIT does not maintain capacity for aerobic energy production and metabolic pathways in the luteal phase.***A*, gene set enrichment analysis of biological processes. *B*, gene set enrichment analysis of cellular compartments. Panels *A* and *B* both analyzed with clusterProfiler (v. 4.6.2). “All” on the x-axis refers to analysis of the main effect of training (i.e., based on combined analysis of all participants irrespective of group). Terms are selected based on biological relevance, and full analysis is available in Supplementary data S3. *C*, boxplots showing median log2fold changes of extracellular matrix organization proteins. *D*, Mean log2fold changes of extracellular matrix organization proteins. *E*, mean log2fold changes in mitochondrial protein complex abundances. Proteins were annotated with MitoCarta 3.0. *F*, mean log2fold changes in abundances of proteins annotated to gene ontology terms related to fatty acid oxidation, glycolysis, and tricarboxylic acid cycle. *p*-values above bars are within-group and between treatments are between-group (group × time interaction effect). FB, follicular phase-based training; LB, luteal phase-based training.

Gene set enrichment analysis was conducted with the R package clusterProfiler (34) (v. 4.4.4). All quantified proteins (total number: 4155) were ranked according to log2fold change, and enrichment was assessed against gene ontology categories (GO: Biological Process and Cellular Component) using the clusterProfiler::GSEA. function. Gene sets were tested for enrichment at the extremes of the ranked list without applying a hard significance threshold. *p*-values were adjusted for multiple testing using the Benjamini-Hochberg procedure. Gene set enrichment analysis was conducted on within-group changes for FB and LB, as well as on the main effect of time changes (“All” in Fig. 2, *A* and *B*; based on limma output in Supplementary data S2, sheet 1 (Main effect of time)).

#### Comparison of mean protein Abundances

Comparison of mean protein abundances of selected biological GO-terms (e.g., cytosolic ribosomal proteins) was performed using the rstatix R package (v.0.7.2). All proteins annotated to the selected term were averaged and within-group changes (pre vs. post) were evaluated using paired t-tests, while between-group differences in log_2_ fold change were tested using unpaired t-tests. *p*-values were reported alongside estimated mean differences and 95% confidence intervals.

## Results

### Participants Exhibited Regular Menstrual Cycles and Trained in the Correct Phases

To verify phases before and throughout the study, we employed a comprehensive inclusion process. Utilizing these criteria, we initially screened 82 females of which 49 fulfilled the eligibility criteria for inclusion (Fig. 1*C*). Of these, 33 completed the training intervention and of these eight were excluded due to serum progesterone concentrations <16 nmol L^−1^ (FB n = 4, LB n = 4; Fig. 1*D*). Thus, the final analysis is based on the remaining 25 females (FB n = 13, LB n = 12) (Table 1) with confirmed cycle lengths of follicular and luteal phases of equal duration (Fig. 1*E*). Although cycle length varies, our included females exhibited little variation in phase length (follicular phase: 1 ± 1 (mean ± SD) day; luteal phase: 1 ± 1 day) and day of ovulation (1 ± 1 day).

**Table 1:** Characteristics of the participants in the follicular phase-based training (FB) and luteal phase-based training (LB) groups

	FB (n = 13)	LB (n = 12)	All (n = 25)
Age (years)	28 ± 3	29 ± 5	28 ± 4
Weight (kg)	60.3 ± 6.1	69.9 ± 7.7	64.9 ± 8.4
BMI	22.1 ± 1.0	24.5 ± 2.1	23.3 ± 2.0
Fat percent *(%)*	22.8 ± 3.2	23.8 ± 4.2	23.2 ± 3.8
$\dot{V}$ O_2max_ (mL·min^−1^)	3107 ± 328	3485 ± 472	3288 ± 439
$\dot{V}$ O_2max_ (mL·min^−1^ kg^−1^)	51.7 ± 3.5	49.9 ± 4.1	50.8 ± 3.9
Training level (hours·week^−1^)	5.9 ± 2.8	7.2 ± 4.1	6.5 ± 3.5

BMI, body mass index; $\dot{V}$ O_2max_, maximal oxygen consumption. Data are presented as mean ± SD.

### Menstrual Cycle Phase-Based SIT Induces Differential Muscle Proteomic Remodeling

To comprehensively decipher protein-wide adaptations in skeletal muscle in response to phase-based SIT, we utilized a state-of-the-art proteomics workflow (Fig. 1*F*). We identified ∼4300 proteins per sample (Fig. 1*G*, 4155 after filtering for 70% valid values) with a dynamic range spanning five orders of magnitude (Fig. 1*H*). Notably, the correlation of the log2fold change of each protein was low between groups (Fig. 1*I*), demonstrating a notable divergence in muscle adaptation with phase-based training.

Among the proteins with the highest increase in abundance were proteins such as SERPINH1 and PCOLCE (both involved in the biosynthetic pathway of collagen (35, 36)), COL5A2 (structural component of group 1 collagen), FTL (involved in iron homeostasis), and S100A13 (a member of the calcium-binding S100 super family) (Fig. 1*J*) suggesting adaptations in tissue remodeling and cellular signaling pathways across both phase-based training interventions. Among the greatest decreases in abundance were several proteins related to mitochondrial function and biogenesis, consisting of cytochrome c oxidase subunit 7 (COX7C), an oxidative phosphorylation complex assembly factor (HIGD2A (37)), and a mitochondrial pyruvate carrier (MPC1). In addition, two proteins related to smooth muscle cell contraction were among the most downregulated (SMTNL1 (38) and CNN1 (39)).

At the individual protein level, few proteins achieved statistical significance with a stringent FDR cutoff of 5%. In FB, no individual proteins reached statistical significance. In LB, seven proteins were significantly increased (MVP, SNRPF, SAMHD1, STAT1, SERPINH1, RTN4, and GPR89A), whereas two proteins were significantly decreased (IAH1 and SOD3).

To assess whether baseline differences in body weight, $\dot{V}$ O_2max_, and weekly training volume influenced the observed proteomic changes, we repeated our analysis with these factors included as covariates in the linear model. While the number of significantly regulated proteins varied slightly (notably in LB), gene set enrichment results for key pathways (e.g., electron transport chain, TCA cycle) remained largely unchanged. The results with inclusion of covariates and comparison to non-covariate results are presented in Supplementary data S5. Full tables of differential expression and gene set enrichment analyses with covariate adjustment are provided in Supplementary data S2 and S3.

### Luteal Phase-Based Training Does Not Maintain Mitochondrial Protein Abundance or Proteins of the Tricarboxylic Cycle

Although few individual proteins met a stringent FDR threshold of 5%, we observed consistent enrichment for coordinated biological processes by gene set enrichment analysis within each group and on the combined data independent of group (“all” in Fig. 2, *A* and *B*). Surprisingly, the most significant changes were terms related to mitochondrial energy production, in which luteal phase-based training appeared to exhibit a pronounced depletion, which was paralleled by depletion in proteins related to the tricarboxylic acid cycle (Fig. 2, *A* and *B*). On the other hand, both groups exhibited enrichment in terms related extracellular matrix organization (Fig. 2*B*), particularly proteins of the major collagen types, were upregulated in both groups (Fig. 2*C*) with a greater increase with follicular phase-based training (Fig. 2*D*).

To unfold the enrichment analysis findings, we assessed all proteins belonging to a mitochondrial complex of the electron transport chain (annotated with MitoCarta 3.0). Here, we were surprised to note a consistent decrease in the abundance of proteins belonging to complex I-IV of the electron transport chain with luteal phase-based SIT (Fig. 2*E*), suggesting impaired mitochondrial energy production and metabolic efficiency, which coincides with the observed decrease in $\dot{V}$ O_2max_. Distinct responses between follicular and luteal phase-based training were also evident for proteins of the tricarboxylic acid cycle, and to a lesser extent, fatty acid oxidation (Fig. 2*F*), highlighting the distinct metabolic adaptation to phase-based training. However, proteins of the glycolysis pathway were seemingly unaffected by the group. And while mitochondrial and TCA pathways were detrimentally affected in the LB group, cytosolic ribosomal proteins exhibited slight increases in abundance, which was favorable towards luteal phase-based training.

### Superior Outcomes in Cardiorespiratory Fitness With Follicular Phase-Based High-Frequency SIT

**Fig. 3:**
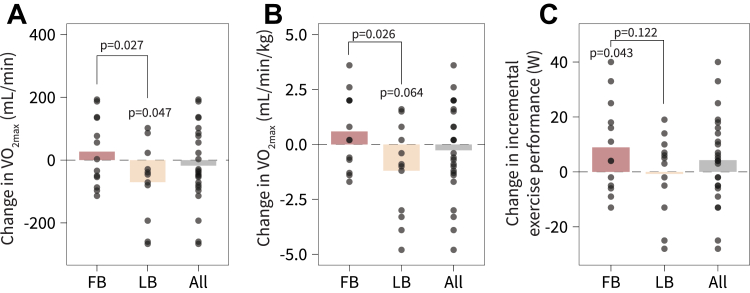
**High-frequency phase-based sprint interval training (SIT) in the follicular phase (FB) and luteal phase (LB) induces differential outcomes in maximal oxygen consumption and incremental exercise performance.***A*, change in maximal oxygen uptake ($\dot{V}$ O_2max_). *B*, change in relative $\dot{V}$ O_2max_. *C*, change in incremental exercise performance. Data are presented as means with individual changes. *p*-values above bars are the within-group effect. *p*-values between treatments are between-group (group × time interaction effect). FB, follicular phase-based training; LB, luteal phase-based training.

The differential adaptations in the global proteome to menstrual cycle phase-based SIT were also observed at a full body level. FB was superior to LB in terms of adaptations pertaining to cardiorespiratory fitness (group × time interaction: *p* = 0.027) (Fig. 3, *A* and *B*). While $\dot{V}$ O_2max_ declined by 117 ml∙min^−1^ in LB (95%CI -204 to −29, *p* = 0.047), corresponding to a 3% decline, it was maintained in FB (*p* = 0.364). In addition, only FB improved their exercise capacity with the intervention (95% 0.3–18 W, *p* = 0.043) (Fig. 3*C*).

## Discussion

Herein, we utilized MS-based proteomics and a strict set of inclusion criteria with longitudinal monitoring in a cohort of eumenorrheic well-trained females to uncover protein wide adaptations in skeletal muscle in response to high-frequency follicular- or luteal phase-based SIT. While SIT, in general, enriched ribosomal content, we observed some notable divergent adaptations between the phase-based training groups. Specifically, we show that luteal phase-based SIT elicits pronounced dilution of the mitochondrial pool and downregulation of tricarboxylic acid cycle proteins, and on the whole-body level, a decrease in cardiorespiratory fitness. In contrast, follicular phase-based training elicited superior adaptations in extracellular matrix reorganization and maintenance of the major metabolic pathways. These findings provide intriguing indications that exercise responsiveness may be heightened during the high frequency training sessions performed in the follicular phase of the menstrual cycle in well-trained females.

SIT is a widely utilized training form because of its demonstrated efficacy to enhance performance in the severe intensity domain in already well-trained individuals and its efficiency to promote beneficial cardiovascular and mitochondrial adaptations within only a few weeks in sedentary individuals (40, 41, 42). Here, we provide the first in-depth proteome analysis of the protein-wide response to SIT in skeletal muscle of a female athlete cohort. Despite the already trained nature of the included cohort, we show that SIT triggers pronounced upregulation of ribosomal and extracellular matrix proteins, likely in response to increased demands for protein synthesis and structural integrity (43, 44). In addition, we observed pronounced differences in energy production pathways depending on high-frequency training distributions to the follicular or luteal phase. This was particularly evident for proteins of the oxidative phosphorylation complexes, which exhibited a pronounced decrease with luteal phase-based training for all complexes (CI-CV). This was paralleled by a consistent suppression of proteins related to the tricarboxylic acid and lipid metabolism, of which PLIN2 was the most repressed protein. In contrast, follicular phase-based training maintained oxidative phosphorylation complex abundance and metabolic pathways, demonstrating apparent phase-based differences in muscle metabolic adaptation to SIT. Follicular phase-based training also showed significantly greater remodeling of extracellular matrix proteins (especially the major collagen types as for example; COL3A1, COL4A1-3, COL5A3, and COL8A1), which was paralleled by the molecular chaperone SERPINH1—required for collagen protein folding (35)—as one of the most increased proteins.

The suppression of mitochondrial pathways for luteal phase-based training was associated with a ≈3% impairment of $\dot{V}$ O_2max_, whereas it was not only maintained with follicular phase-based training but also paralleled by improved exercise capacity during the ramp test. Although the decline in $\dot{V}$ O_2max_ for luteal phase-based training may appear small, this is a meaningful change for highly-trained individuals (45) and within the error margin of 2 to 3% in our lab (within-subject CV for ramp exercise capacity and $\dot{V}$ O_2max_). In other trained cohorts subjected to SIT, typically males, $\dot{V}$ O_2max_ generally remains unchanged (40), as also observed in the follicular phase-based training group. While one could speculate that preceding training load influenced the performance measurements in our study, this is unlikely considering the recovery from the last training until measurements, as shown in other athlete cohorts (46, 47). SIT is well-tolerated even in untrained individuals (48), and high-frequency training leading up to ramp testing does not influence $\dot{V}$ O_2max_ measurements in well-trained individuals (49). Our results are also coherent across the physiological outcomes and muscle proteome adaptations, which collectively suggest that follicular phase-based SIT is superior to luteal phase-based SIT under the settings in question.

Our findings highlight the menstrual cycle phase as an important physiologic confounder for muscle adaptations to exercise training and suggest phase-specific training as a potential strategy in optimizing training schedules for female athletes. This should not discourage female inclusion in sports science research. Rather, it emphasizes the imperative for further investigation. Implementing phase-based SIT would require careful tracking of each athlete’s menstrual cycle, with the understanding that certain individuals exhibit more pronounced within-subject differences in both hormone fluctuations and length of the menstrual cycle (17, 50). Nevertheless, given that pronounced muscle remodeling and phenotypic changes apparently occur after one cycle of phase-based SIT, this should encourage follow-up studies over several cycles where an even greater response could be expected.

## Data Availability

The mass spectrometry proteomics data have been deposited to the ProteomeXchange Consortium via the PRIDE (51) partner repository with the dataset identifier PXD051852.

## Supplementary Data

This article contains supplementary material.

## Additional Information

Correspondence and requests for material should be addressed to MH.

## Conflict of interest

The authors declare that they have no conflicts of interest with the contents of this article.

## Supplementary data

Supplementary data 1 Dataset. Table 1: Data set containing log2-transformed, median scaled intensity values for all quantified proteins (prior to filtering for 70% valid values). Table 2: Data set containing log2-transformed, median scaled intensity values for all quantified proteins after filtering. Table 3: Design matrix with sample metadata for limma analysis.

Supplementary data 2 Results. For each result sheet is presented the protein name, log2fold change, raw *p*-value, adjusted *p*-value (q-value), the number of unique peptides used to identify the protein as mean across all samples, and the UniProt accession identifier. Table 1: Result sheet for main effect of time (post - pre, independent of group). Table 2: Results sheet for FB group (post – pre). Table 3: Results sheet for LB group (post – pre). Table 4: Results sheet for interaction effect (time × group). Table 5: Results sheet for FB-group (with covariate “weight”; post – pre). Table 6: Results sheet for FB-group (with covariate “$\dot{V}$ O_2max_”; post – pre). Table 7: Results sheet for FB-group (with covariate “training h/w”; post – pre). Table 8: Results sheet for LB-group (with covariate “weight”; post – pre). Table 9: Results sheet for LB-group (with covariate “$\dot{V}$ O_2max_”; post – pre). Table 10: Results sheet for LB-group (with covariate “training h/w”; post – pre).

Supplementary data 3 Gene set enrichment analysis results. Table 1: Complete results for gene set enrichment analysis (GO:BP). Table 2: Complete results for gene set enrichment analysis (GO:CC). Table 3: Complete results for gene set enrichment analysis (with covariates; GO:BP). Table 4: Complete results for gene set enrichment analysis (with covariates; GO:CC).

Supplementary data 4 Participant and performance data.

Supplementary data 5 Inclusion of covariates in limma model.
