# Supplementary material for: Global Proteomics Reveals Distinct Muscle Adaptations to Menstrual Cycle Phase-Based Sprint Interval Training in Endurance-Trained Females
Source: Mol Cell Proteomics. 2025 Aug 14;24(10):101053. doi: 10.1016/j.mcpro.2025.101053 (PMC12538072; doi:10.1016/j.mcpro.2025.101053)
Supplement: Supplementary data 5 — Inclusion of covariates in limma model. [file mmc5.docx]

Supplementary data 5: Inclusion of covariates in limma model

*Differential expression analysis:*

We included variables in which groups differed at baseline (body weight, V̇O_2max_, and weekly training hours) as covariates in the linear models. Using a false discovery rate (FDR) of <5%, no proteins were significantly regulated in the FB group irrespective of covariate inclusion. In contrast, while 9 proteins were significantly regulated in the LB group with training, this was reduced by covariate inclusion of weekly training hours (6 proteins), V̇O_2max_ (1 protein) and body weight (1 protein).


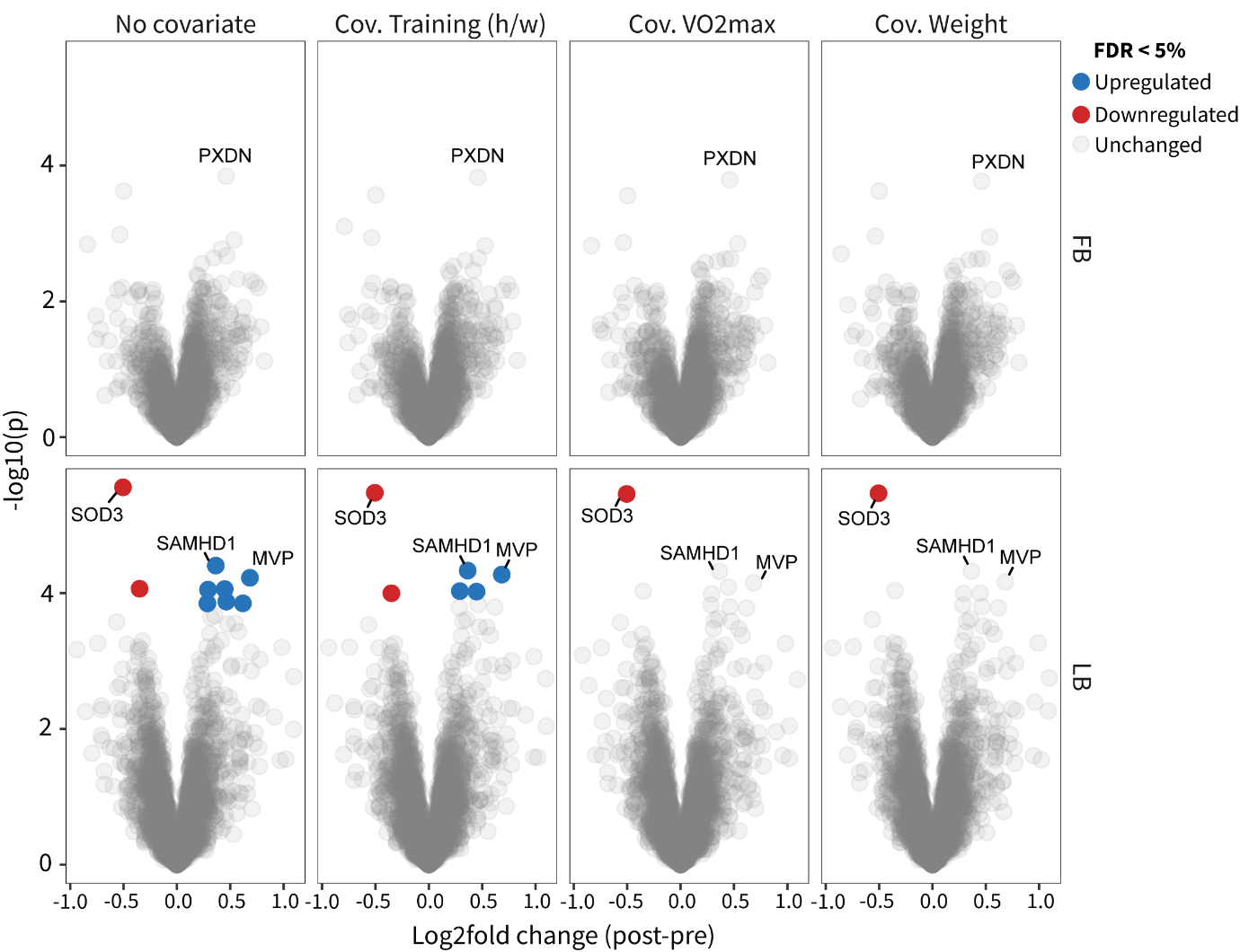


Figure 1. Volcano plots showing differential expressions in each intervention group with and without covariates included in the limma model. P-values adjusted with q.values as described by Storey and Tibshirani (PMID: 12883005).

*Correlations:* Despite a numerical change in the number of regulated proteins in the LB group, overall correlations between results with and without covariates were high (Figure 2). The maximum difference in log2fold change with the intervention between results with and without covariate inclusion was 0.056.


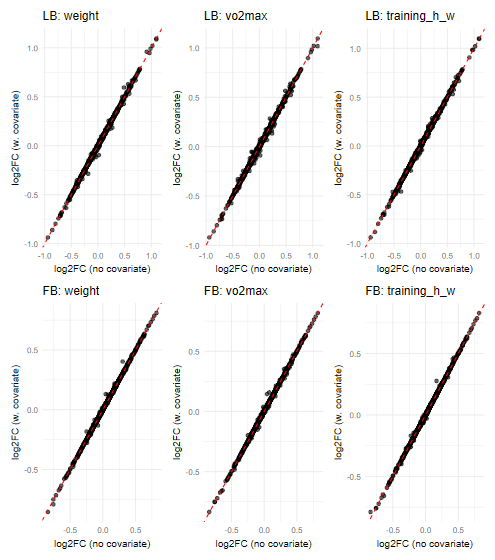


Figure 2: Model comparisons. Plot showing correlations between log2fold changes with different covariates included in the limma model (weight, V̇O_2max_, and weekly training hours) vs. log2fold changes without covariates for each intervention group. Red dashed line is identity line.

*Gene set enrichment analysis:*

When conducting gene set enrichment analyses on the results with and without inclusion of covariates in the linear models, enrichment results were largely unaffected (Figure 3). For example, “electron transport chain” was depleted in LB despite inclusion of covariates (weight: p = 5.5E-08, V̇O_2max_: p = 2.7E-08, weekly training hours: p = 7.8E-08) and the same was true for “tricarboxylic acid cycle enzyme complex” (weight: p = 0.040, V̇O_2max_: p = 0.035, weekly training hours: p = 0.038).


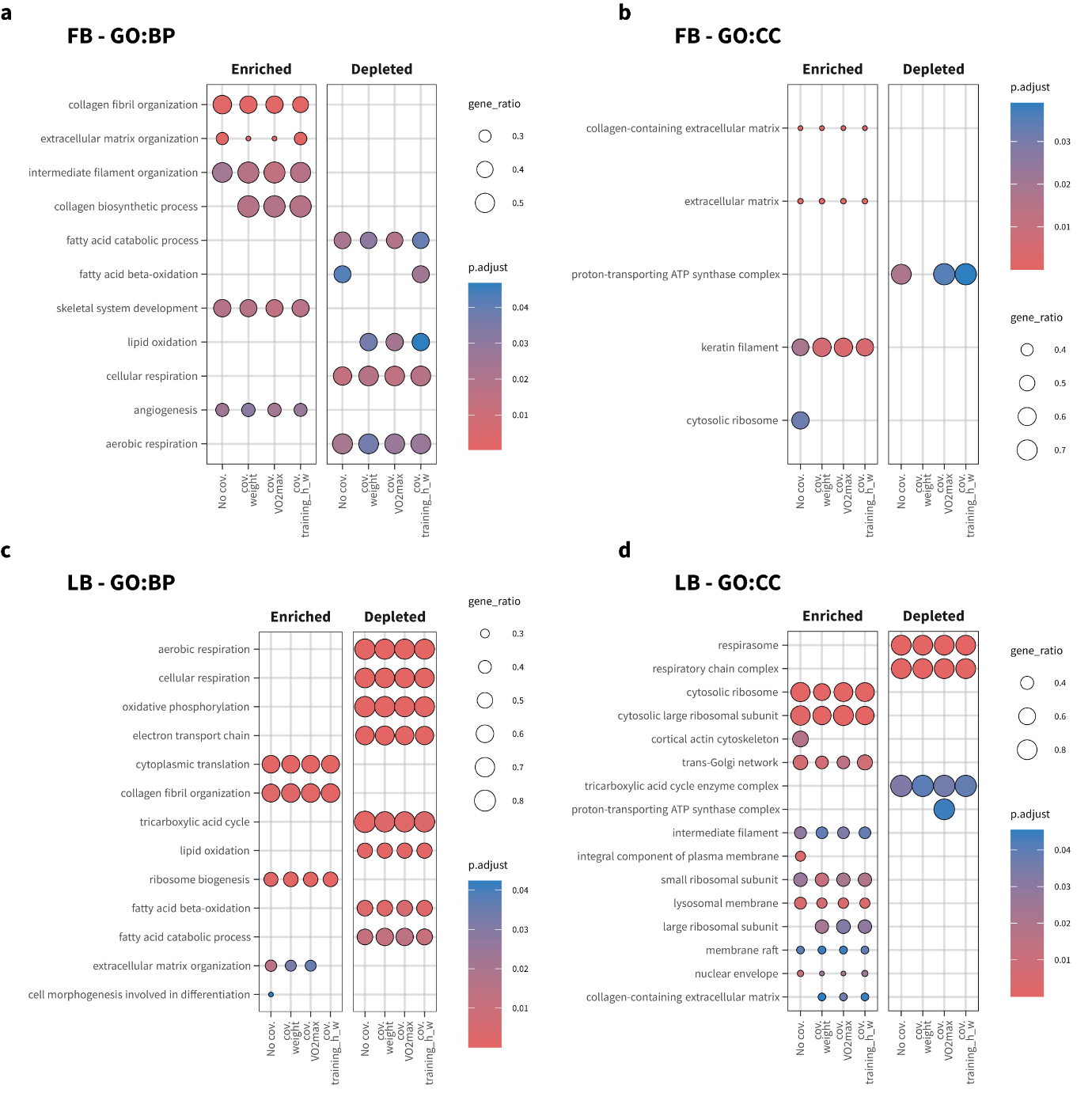


Figure 3. Gene set enrichment analyses with and without inclusion of covariates. **a**, follicular phase-based training group (FB), gene ontology biological process (GO:BP) terms. **b**, FB group, gene ontology cellular compartment (GO:CC) terms. **c,** luteal phase-based training group (LB) GO:BP terms. **d**, LB GO:CC terms.
